# Photolytic Controlled Release Formulation of Methotrexate Loaded in Chitosan/TiO_2_ Nanoparticles for Breast Cancer

**DOI:** 10.3390/ph15020149

**Published:** 2022-01-26

**Authors:** Nusaiba Al-Nemrawi, Fatima Hameedat, Belal Al-Husein, Sukaina Nimrawi

**Affiliations:** 1 Department of Pharmaceutical Technology, Faculty of Pharmacy, Jordan University of Science and Technology, Irbid 22110, Jordan; fjhmeedat13@ph.just.edu.jo; 2 Department of Clinical Pharmacy, Faculty of Pharmacy, Jordan University of Science and Technology, Irbid 22110, Jordan; belalhusein@just.edu.jo; 3 Faculty of Pharmacy, Zarqa University, Zarqa 13132, Jordan; sukainanimrawi@yahoo.com

**Keywords:** TiO_2_ nanoparticles, breast cancer, chitosan nanoparticle, methotrexate, UV light

## Abstract

A new system composed of chitosan nanoparticles loaded with methotrexate (MTX-CS-NPs) and functionalized with photocatalytic TiO_2_ nanoparticles (TiO_2_-NPs) was prepared. This system is expected to initiate polymeric rupture of MTX-CS-NPs and subsequently release MTX, upon illumination with UV light. MTX-CS-NPs were prepared and characterized in terms of particle size, charge, polydispersity and drug release before and after coating with TiO_2_-NPs. The release of MTX in vitro was studied in dark, light and UV light. Finally, coated and uncoated MTX-CS-NPs were studied in vitro using MCF-7 cell line. The functionalized NPs were larger in size, more polydisperse and carried higher positive charges compared to the unfunctionalized NPs. The entrapment efficacy was high reaching 75% and was not affected by coating with MTX-CS-NPs. Further, less than 5% of methotrexate was released after 80 h from uncoated NPs and the release was not enhanced by UV illumination of the particles. In contrast, the release from functionalized NPs was enhanced, reaching 40% after 80 h, as the particles were stroked with UV light and as the amount of TiO_2_-NPs used in coating increased. Finally, coating the MTX-CS-NPs with TiO_2_-NPs significantly enhanced their cytotoxicity on MCF-7 cells. The coated MTX-CS-NPs recorded low cell viabilities compared to the other formulations. In conclusion, the drug release of MTX-CS-NPs could be triggered and controlled remotely by coating with TiO_2_-NPs, which maybe more effective in cancer treatment.

## 1. Introduction

Methotrexate (MTX) is a stoichiometric inhibitor of dihydrofolate reductase, which is essential for DNA synthesis. MTX is a chemotherapeutic agent used for treating many types of cancer cells that overexpress folate receptors on their surfaces such as leukemias, breast cancer, head & neck cancer, lymphomas and carcinomas [1]. However, increase in drug export from cells and impairment of drug import into cells may increase the cells resistant to MTX. Furthermore, when MTX is locally administered in a soluble form, it is rapidly absorbed through capillaries into the circulatory system, and hence, rapidly excreted [2,3]. All of these reasons contribute to therapeutic failure of MTX treatment. Therefore, many researchers have proposed the encapsulation of MTX in polymeric nanoparticulate systems to enhance its duration, alter its pharmacokinetic behavior within tumor cells and to control the drug release. Many polymers have been used for that purpose, including chitosan [4,5].

Chitosan (CS) is a natural, linear polysaccharide that have been used in preparing nanoparticles (NPs.) CS has been used in pharmaceutical and biomedical applications due to its unique properties such as biocompatibility, biodegradability, non-toxicity and bioadhesive characteristics. CS nanoparticles (CS NPs) have been used as a colloidal drug carrier for targeted delivery to specific sites, as well as, for gene and vaccine delivery and cancer therapy [6,7]. CS NPs have been prepared by different methods. It was reported that the method used in preparing the NPs, the parameters and the starting materials properties affect the prepared NPs physicochemical properties and the drug release profile. Ionic gelation technique is one of the most widely used method in preparing CS NPs. It is based on the ionic interaction between the amino groups of chitosan and a crosslinker [8,9]. MTX loaded in CS NPs have been developed previously in order to improve and control the drug delivery to tumors. Further, covalently conjugated CS and MTX has also been reported to deliver MTX [5,10].

One of the important drawbacks of polymeric NPs is the drug release at a predetermined rate that is irrespective of patient needs or physiological circumstances. A triggerable drug delivery system should allow timing-control of the therapeutic effect. This on-demand drug release from nanoparticles maximize tumor killing and minimize metastatic spread [11,12]. 

In general, the drug release from polymeric nanoparticles depends on diffusion through the polymers. To provide on-demand drug release from NPs, these NPs could be functionalized to be responsive to specific triggers, such as pH, temperature, redox, proteins, light and magnetic field [13,14,15,16]. There are a significant number of photo-controlled release systems that are based on noncovalently assembled polymeric systems or on cyto-incompatible shortwave UV light [17,18,19].

One of these materials is titanium dioxide nanoparticles (TiO_2_-NPs). Adding TiO_2_-NPs to CS NPs may allow on demand drug release by photolytic degradation. In previous researches, Cs/PVA blend was applied as a nanoreactor for Ag and Au nanoparticle and showed promising anticancer applications [20]. Other researchers found that triggering hydrophilic polymers such as chitosan could control drug release in response to UV irradiation [21,22]. 

Titanium dioxide (TiO_2_), a natural mineral oxide, exists in different forms such as anatase, rutile, and brookite forms. Titanium dioxide nanoparticles (TiO_2_-NPs) represent a new type of inorganic chemical material that is widely used in cosmetics, pollution treatment, food preservation, pharmaceuticals and painting field. More recently, TiO_2_-NPs have been used in biomaterials due to their high stability, antimicrobial and anticorrosive properties [23]. TiO_2_-NPs have unique photocatalytic properties that has led to extensive research on their potential use as a disinfectant, antibiotic, biological sensor and tumor cell killing agent [24]. TiO_2_-NPs were found to induce apoptosis and oxidative stress on cells, both of human and animal origin, studied in vitro and in vivo. This stress was found to cause a sequential events of signal transduction that lead to accumulation of P53, a tumor suppressor protein, which block the cellular proliferation [25].

Recently, the combination of more than one strategy in cancer treatment have been explored to promote synergism among the different drugs against cancer cells and suppresses drug resistance through distinct mechanisms of action. Further, combined therapy were found to enhance therapeutic effectiveness and reduces side effects of the drugs by improving their pharmacokinetics [26]. Therefore, in this research TiO_2_-NPs were used with MTX-CS-NPs as cancer therapeutics. Further, TiO_2_-NPs were used to trigger the drug release from MTX-CS-NPs upon the use of UV light. In this system, TiO_2_-NPs are used to initiate the rupture of the polymeric bonds of CS NPs, when exposed to light. 

In this study, a new formulation of MTX loaded in chitosan nanoparticles and coated with TiO_2_-NPs was prepared. MTX-CS-NPs were characterized in terms of particle size, zeta potential, polydispersity index and drug release in vitro. Further, the coated MTX-CS-NPs were characterized and compared to the uncoated NPs using Fourier transform infrared (FTIR) spectroscopy and X-ray diffraction (XRD) and dye tests. The release of MTX in vitro from the coated NPs was studied in the dark and under visible light and UV light. Finally, MTX-CS-NPs were studied in vitro using the human breast cancer tumor cell line MCF-7. In this work we expect that combining TiO_2_-NPs with MTX-CS-NPs may be more effective for cancer treatment. 

## 2. Results

### 2.1. Characterization of MTX-CS-NPs Coated with TiO_2_-NPs

MTX-CS-NPs were prepared in this work using an ionic gelation method and coated with TiO_2_-NPs. The mean particle size, PDI, zeta potential and the EE% of both uncoated and coated MTX-CS-NPs are summarized in Table 1. The uncoated MTX-CS-NPs were smaller in size than coated NPs. The higher size of the coated NPs proves the interaction between the NPs and TiO_2_-NPs and indicates that TiO_2_-NPs attached themselves to the surfaces of MTX-CS-NPs. Even though the sizes of the particles are somehow large, they still can be used in breast cancer therapy. Due to the enhanced permeability and retention (EPR) effect of carcinogenic tissues, it was reported that polymeric nanoparticle in a range of 100–400 nm are suitable to be used in targeting carcinogenic tissues [27]. All the NPs prepared in this work carried a positive charge, where the charges become more positive once the NPs were coated with TiO_2_-NPs. 

The PDI is a measure of the heterogeneity of a sample based on size. Polydispersity can occur due to the size distribution of particles in the sample or to their agglomeration during analysis [28]. The International Standards Organization (ISO) has established that samples with PDI values less than 0.05 are monodisperse, while samples with PDI values more than 0.7 are polydispersed [29]. Therefore, as the value of PDI is closer to zero, the sample is more homogeneous and the aggregation of NPs is minimum. On the other hand, when the value is closer to one, the sample may contain polydispersed particles or the aggregation is high. Herein, the PDI of both coated and uncoated NPs implies monodispersity (less than 0.7). Coated MTX-CS-NPs had higher PDI values suggesting lower monodispersity, which may be related to the irregular attachment of TiO_2_-NPs on the surfaces of MTX-CS-NPs upon coating.

An effective formulation that is safe, stable and efficient requires certain limits of homogeneity of NPs for many reasons. Firstly, it has been reported that heterogenicity enhances the sedimentation of particles. Therefore, reducing the heterogenicity is expected to stabilize the samples when suspended for longer periods of time [30]. Secondly, the distribution of particles’ sizes affects the drug release kinetics, which affects the drug bioavailability [31]. Thirdly, uniformity of any patch affects its flowability, compressibility and many others industrial variables. Finally, monodisperse NPs were proven to facilitates their cellular uptake [31].

The EE of all preparations was almost the same and was not affected by coating. Similar results were reported previously by other researchers, where coating or loading NPs in films do not affect the EE of drug in NPs [32,33].

**Figure 1:**
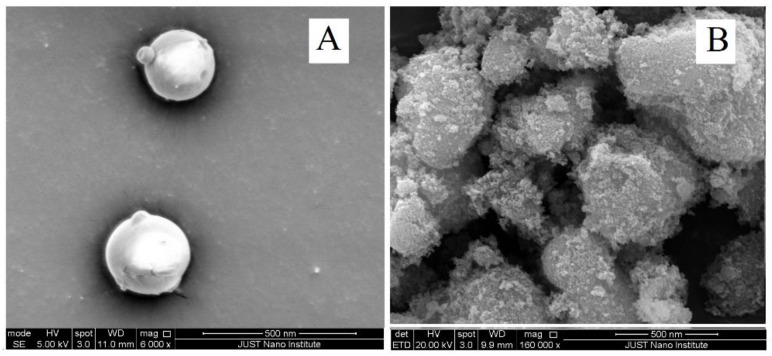
SEM image of MTX-CS-NPs (**A**) uncoated NPs, (**B**) coated NPs with TiO_2_-NPs (F_1_).

Figure 1 shows a representative SEM image of the uncoated particles (A) and uncoated particles of F_1_ (B). The uncoated MTX-CS-NPs prepared in this work showed spherical particles that are well separated from each other. On the other hand, the coated particles are wrinkled, with a fairly spherical morphology, as confirmed by SEM. The coated particles’ surface is rough and show smaller particles attached to it, which may be the precipitated TiO_2_-NPs on MTX-CS-NPs. 

**Figure 2:**
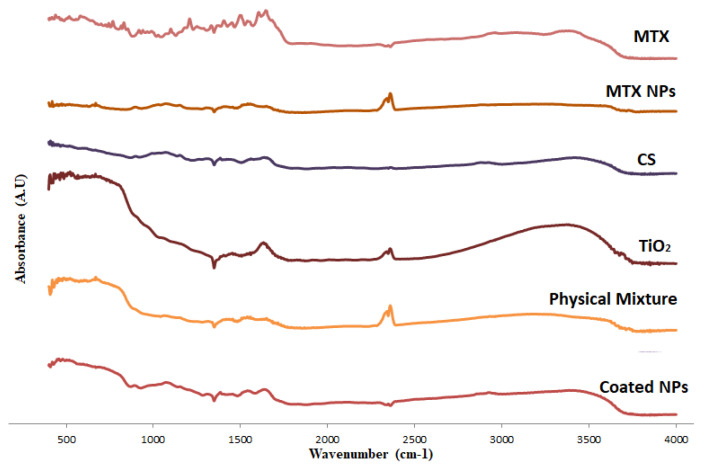
FTIR spectra of CS, MTX, TiO_2_ NPs, CS-MTX NPs, the physical mixture (MTX-CS-NPs with TiO_2_ NPs) and MTX-CS-NPs coated with TiO_2_ NPs.

FTIR results for CS, MTX, TiO_2_-NPs, MTX-CS-NPs, the physical mixture (MTX-CS-NPs with TiO_2_-NPs) and MTX-CS-NPs coated with TiO_2_-NPs are shown in Figure 2. First of all, the spectra of CS, MTX and MTX-CS-NPs were compared to detect the formation of CS-MTX NPs. Then the spectrums of TiO_2_-NPs, MTX-CS-NPs, the physical mixture and the coated MTX-CS-NPs were compared to explore the coating of MTX-CS-NPs with TiO_2_-NPs.

For pure CS, a characteristic band was observed at 3447 cm^−1^ related to −NH_2_ and −OH groups stretching in chitosan. Further, a band corresponding to amine stretching at 1110 cm^−1^ was also noticed. The N-H bending in amine group at 1504 cm^−1^ and at 1573 cm^−1^, C-H bending at 870 cm^−1^, can be noticed [34]. 

For pure MTX, FTIR spectral analysis showed the characteristic peaks at 1643 cm^−1^ related to -CO-NH group, 1543 cm^−1^ and 1500 cm^−1^ related to the aryl system and 830 cm^−1^ that is related to an aromatic ring system [35]. This spectrum confirms the purity of the drug used in preparing the MTX-CS-NPs. For MTX-CS-NPs, the decrease in intensity of the band related to primary NH_2_ groups in CS spectrum indicate that there is an interaction between CS and MTX. Further, the disappearance of the characteristic peaks of MTX; 1643 cm^−1^ and 830 cm^−1^ and the shifting of other peaks from 1543 cm^−1^ to 1556 cm^−1^ in MTX-CS-NPs spectrum proof the formation of the NPs [36,37].

For TiO_2_-NPs, the FTIR spectrum clearly shows two characteristic bands. The first band is the broadest and is observed at 3500 cm^−^^1^ that corresponds to the stretching of O-H groups. The second band is observed around 1630 cm^−^^1^ and corresponds to bending modes of water in Ti-OH [38,39].

The FTIR spectrum of the physical mixture of MTX-CS-NPs and TiO_2_-NPs exhibited differences from that of the coated NPs. The following major differences were observed: First, the wide peak at 3500 cm^−^^1^ in TiO_2_-NPs spectrum get shallower in the coated NPs but remained the same in the physical mixture. Second, the band observed around 1630 cm^−^^1^ in TiO_2_-NPs spectrum disappeared in the spectrum of the coated NPs. Third, the peak at 2368 cm^−^^1^ in MTX-CS-NPs disappeared but can be noticed clearly in the physical mixture. Finally, many peaks get shifted in the coated NPs but not in the physical mixture. These differences in the spectra of the coated NPs and the physical mixture give a clear proof that TiO_2_-NPs deposited chemically on the surface of MTX-CS-NPs during the coating process. 

XRD was carried out for CS, MTX, TiO_2_-NPs, the physical mixture (MTX-CS-NPs with TiO_2_-NPs) and the coated MTX-CS-NPs with TiO_2__-_NPs. As in FTIR, the spectra of CS, MTX and MTX-CS-NPs were compared to proof the formation of CS-MTX NPs. Then the spectrums of TiO_2_-NPs, MTX-CS-NPs, the physical mixture and the coated MTX-CS-NPs were compared to poof the coating of MTX-CS-NPs with TiO_2_-NPs.

**Figure 3:**
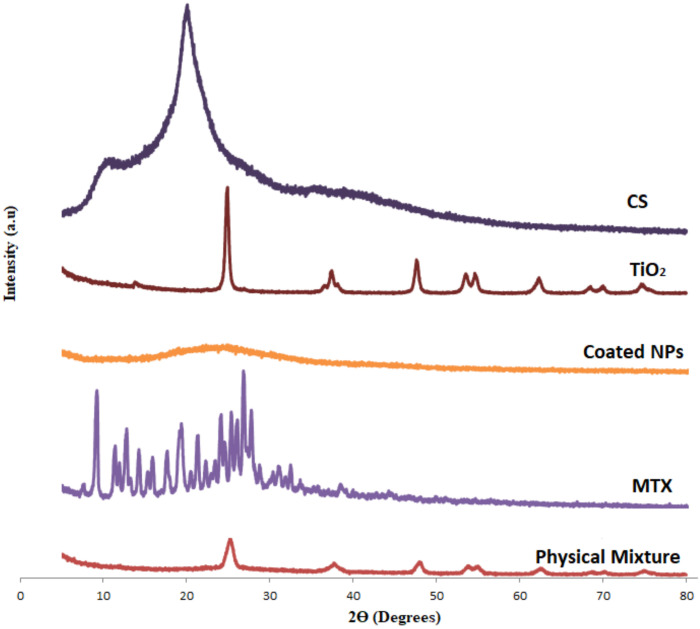
XRD of CS, MTX, TiO_2_ NPs, the physical mixture (MTX-CS-NPs with TiO_2_ NPs) and the coated MTX-CS-NPs with TiO_2_ NPs.

Figure 3 shows that the XRD spectra of MTX have sharp peaks that is related to the crystalline nature of MTX. On the other hand, CS exhibited an amorphous structure that is indicated by its XRD spectra (no peaks). MTX-CS-NPs spectrum has o peaks, where the sharp peaks of MTX disappeared. This indicates the incorporation of MTX within the NPs [9,35]. Further, TiO_2_-NPs exhibited a crystalline structure as indicated by the sharp peaks in its spectrum [38]. When TiO_2_-NPs and MTX-CS-NPs were mixed physically, the peaks of TiO_2_-NPs are still showing up. On the other hand, in the formulation where TiO_2_-NPs and MTX-CS-NPs were allowed to interact, these peaks disappeared indicating chemical deposition of TiO_2_-NPs on MTX-CS-NPs.

**Figure 4:**
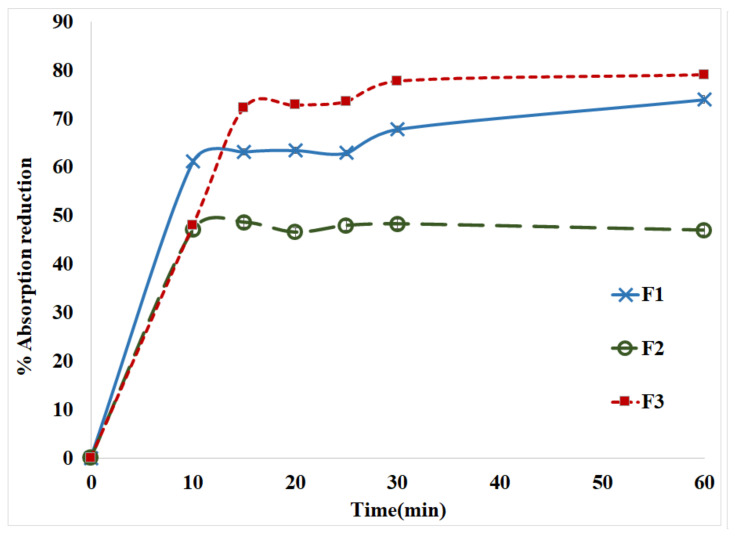
The percentage of absorption reduction for F_1_ (1:1), F_2_ (1:2) and F_3_ (2:1) (the ratio of MTX-CS-NPs to TiO_2_) versus time measured at λmax of 600 nm.

The results of the dye tests for the different formulations are shown in Figure 4. The reduction of the absorption in comparison to a reference was used to measure the amount of TiO_2_-NPs reacted with MTX-CS-NPs. A sample of pure TiO_2_-NPs have been used as a reference in this experiment. For the coated MTX-CS-NPs, as the amount of TiO_2_-NPs reacted increases the absorption decreases, and hence, there is a reduction in the absorption increase. It can be noticed that as the concentration of TiO_2_-NPs increases in the preparation, the reduction in absorption increases. In other words, as the concentration of TiO_2_-NPs increases, the amount reacted with MTX-CS-NPs increases.

### 2.2. In Vitro Drug Release from MTX-CS-NPs Coated with TiO_2_-NPs

**Figure 5:**
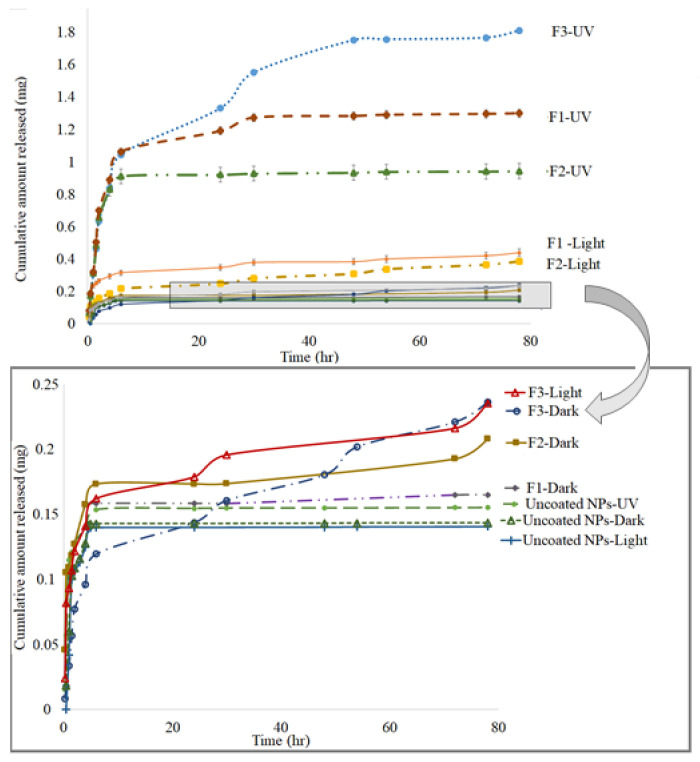
The cumulative amount of MTX released over 72 h from the uncoated MTX-CS-NPs, F_1_ (1:1), F_2_ (1:2) and F_3_ (2:1) (the ratio of MTX-CS-NPs to TiO_2_) in vitro under three different conditions: visible light, dark and UV light.

The cumulative amount of MTX released over 72 h from the uncoated MTX-CS-NPs and coated MTX-CS-NPs (F_1_, F_2_ and F_3_) was investigated in vitro under three different conditions; visible light, dark and UV light. The results are shown in Figure 5.

The release from the uncoated MTX-CS-NPs was not affect by the lightening mode. In general, the release from the uncoated NPs was lower than MTX release from the coated NPs. On the other hand, the release from the coated MTX-CS-NPs was affected by the lighting mode. The highest release was observed when the NPs were illuminated with UV light. The NPs studied in daylight showed lower drug release in comparison to those illuminated with UV light but higher than those studied in the dark. Moreover, when the samples were illuminated with UV light, the highest drug release was from F_3_ followed by F_1_ and the least was from F_2_. When the drug release under daylight was explored, both F_3_ and F_1_ showed almost the same drug release pattern, that was higher than that of F_2_. The lowest drug release occurred for samples kept in the dark, as well as the uncoated samples. These results indicate that encapsulating MTX in CS NPs controlled and sustained the drug release. Further, coating the NPs with TiO_2_-NPs allow faster drug release from MTX-CS-NPs by illuminating the system by UV light. 

### 2.3. Cell Culture

**Figure 6:**
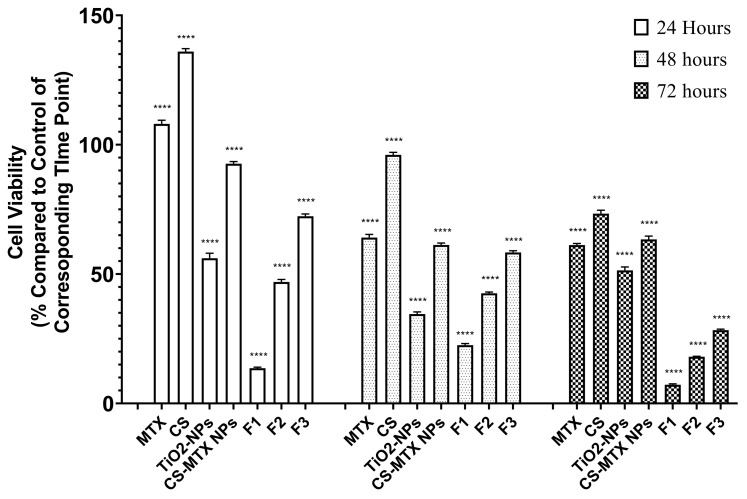
The cell viability of MTX, CS, TPP, TiO_2_ NPs, CS-MTX NPs F1 (1:1), F2 (1:2) and F3 (2:1) (the ratio of MTX-CS-NPs to TiO_2_) on MCF-7 cell line after 24, 48, and 72 h of incubation.Groups were compared using two-way ANOVA. (ns: *p* > 0.05; *: *p* ≤ 0.05; **: *p* ≤ 0.01; ***: *p* ≤ 0.001; ****: *p* ≤ 0.0001).

The cytotoxicity of MTX, CS, TPP, MTX-CS-NPs and coated MTX-CS-NPs (F_1_, F_2_ and F_3_) was studied using an MTT assay on tumor MCF-7 cell line. This test evaluated the mitochondrial function of the cells in a time dependent manner, and herein we studied the cytotoxicity after 24, 48, 72 h of incubation of the cells. Figure 6 shows the cell viability of MCF-7 cell line after 24, 48, and 72 h of incubation with MTX, CS, TPP, TiO_2_ NPs, CS-MTX NPs, F_1_, F_2_ and F_3_. The results showed that the cytotoxicity of MTX-CS-NPs against the tumor cells was greater than that of the free drug and free CS. Free MTX showed almost no cytotoxic effect after 24 h, while moderate cytotoxicity (~60% cell viability) was noticed after 48 h and 72 h. Usually, in cancer researchers the viability assays is performed at 24, 48 and 72 h as a standard procedure. Despite that, 24-h incubation times most of the time don’t show high effects on viability. This behavior of MTX was confirmed by other researchers were free MTX has time-dependent cell toxicity [40]. Pure CS showed no cytotoxicity after 24 h and 48 h, but at the endpoint it showed small cytotoxicity (73.37 ± 1.29 of viability of cells). The cytotoxicity of CS was related previously to the protonation of its amine groups within the components of the biological membrane, which impairs membrane function. The protonated amine can suppress the negative electric charge of the mammal’s cell membranes at physiological pH [41].

Moderate cytotoxicity was observed for uncoated MTX-CS-NPs after 72 h (~60% cell viability). Unlike pure MTX or pure CS, it is important to mention that the toxic effect of the uncoated NPs started from the first point at 24 h. In other words, encapsulating MTX in CS-NPs may not enhance the cytotoxicity of MTX but it affects the pattern and extent of cytotoxicity. 

TiO_2_-NPs significantly reduced the cell growth to 50%. As we mentioned previously, TiO_2_-NPs were found to induce apoptosis and oxidative stress on cells studied in vitro. This stress was found to cause a sequential events of signal transduction that lead to accumulation of P53, which block the cellular proliferation [42].

Coating MTX-CS-NPs with TiO_2_-NPs significantly enhance the cytotoxicity, especially for F_1_, where it the cell viability reached 7% at the endpoint. Further, it can be noticed that the suppression for the three formulations started from the first time point. It worth to mention that the drug release in light, as shown in Figure 5, was higher for F_1_ followed by F_2_, and then F_3_. This order was the same in terms of cytotoxicity on MCF-7 cells, where F_1_ showed the highest cytotoxicity followed by F_2_ and then F_3_.

## 3. Materials and Methods

### 3.1. Materials Used

Low molecular weight chitosan (50 kDa, DDA 90), methotrexate and titanium (IV) oxide (99.5%, <100 nm) were purchased from Sigma-Aldrich (St. Louis, MO, USA). Sodium tripolyphosphate (TPP) was purchased from AZ Chem for Chemicals (Dongguan, Guangdong, China). All other chemicals used in this work are of analytical grade.

### 3.2. Preparation of Chitosan Methotrexate Nanoparticles

MTX-CS-NPs were prepared based on ionic gelation method with a weight ratio of 1:2 between CS and TPP. Two aqueous phases were prepared; the first solution was prepared by dissolving chitosan in 1% acetic acid solution. This solution was mixed gently by magnetic stirrer at room temperature. The second solution was prepared by dissolving TPP and MTX in HPLC water at concentrations of 0.5 mg/mL and 0.7 mg/mL, respectively, and mixed gently by magnetic stirrer at room temperature. After that, the TPP/MTX solution, cooled to 2–4 °C, was added to chitosan solution, heated to 60 °C, by a syringe pump (Next Advance, Troy, NY, USA) at a flow rate of 0.25 mL/min and a speed of mixing ~700 rpm [9]. Half the prepared MTX-CS-NPs was purified using a dialysis bag (cutoff = 12–14 kDa). The dispersion was placed into the dialysis bags and immersed in 100 mL of distilled water for 4 h. After that, the trapped MTX-CS-NPs were freeze-dried at −60 °C overnight. The nanoparticles were stored in tight glass container at 4–8 °C for further use. The other portion of the prepared MTX-CS-NPs was coated with TiO_2_-NPs. 

### 3.3. Coating Chitosan Methotrexate Nanoparticles with Titanium Dioxide

Three weight ratios of MTX-CS-NPs to TiO_2_-NPs were prepared chemically, namely 1:1, 1:2 and 2:1, and were referred to as F_1_, F_2_ and F_3_, respectively. First, TiO_2_-NPs was dissolved in 10 mL of 1% acetic acid solution. The solution was vortex at room temperature, and then, filtrated through a syringe filter (0.22 µm). After that, the TiO_2_-NPs solution was added drop wise to the solution containing MTX-CS-NPs at room temperature by a syringe pump at a flow rate of 2.5 mL/min and a speed of 700 rpm. The final dispersion was purified as mentioned in the previous section using a dialysis bag. Finally, the coated MTX-CS-NPs with TiO_2_-NPs were freeze-dried and stored in tight glass container at 4–8 °C for further use. 

### 3.4. Characterization of MTX-CS-NPs before and after Coating with TiO_2_-NPs

The mean particle size, zeta potential and polydispersity index (PDI) of MTX-CS-NPs and MTX-CS-NPs coated with TiO_2_-NPs were measured using Zetasizer Nano ZS instrument (Malvern Instruments Limited, Malvern, UK) at 25 °C. In addition, the encapsulation efficiency (EE) was calculated using the following equation:

$$\mathrm{EE}\;(\%)=\;\frac{\mathrm{Total}\;\mathrm{MTX}\;\mathrm{amount}-\mathrm{Free}\;\mathrm{MTX}\;\mathrm{amount}}{\mathrm{Total}\;\mathrm{MTX}\;\mathrm{amount}}\;\times \;100\%$$

where the free MTX amount, is the amount of MTX outside the dialysis bags used in the preparation of the NPs. MTX amounts were analyzed using HPLC-UV (Thermo Scientific, Waltham, MA, USA). Briefly, 10 mM KH_2_PO_4_ buffer (pH = 3), methanol and acetonitrile were used to prepare the mobile phase in a ratio of 70:20:10. C_18_ column was used and the flow rate was set at 1 mL/min. 100 µL of each sample was injected and analyzed at λ_max_ of 302 nm at 25 °C. 

Both the uncoated and coated MTX-CS-NPs were pictured using SEM (Thermo Scientific, model: QUANTA FEG 450, Waltham, MA, USA). The samples were coated with carbon film prior to analysis, and then, studied under microscope.

In order to proof the formation of MTX-CS-NPs, CS, MTX, MTX-CS-NPs were compared using FTIR. Further, the FTIR spectra of MTX-CS-NPs coated with TiO_2_-NPs was compared to the spectra of MTX-CS-NPs before coating and the spectra of the physical mixture of MTX-CS-NPs with TiO_2_-NPs. 

FTIR samples were prepared by mixing them with KBr and pressed into pallets for measurements. A Shimadzu IR spectrophotometer (Shimadzu, Kyoto, Japan) with a high-performance diamond single-bounce ATR accessory (wave number 400–4000 cm^−1^, resolution 4 cm^−1^ with 64 scans per spectrum) was used to record the results. 

XRD was used to study the physical structure of the coated MTX-CS-NPs with TiO_2_-NPs in comparison to CS, MTX, TiO_2_-NPs and the physical mixture of MTX-CS-NPs and TiO_2_-NPs. XRD was performed using an Ultima IV X-ray diffractometer (Rigaku, Tokyo, Japan) using cobalt radiation (CuKα) with a voltage of 40 kV and a current of 30 mA at room temperature. The diffraction angles of 2θ starting from 0° to 60° were used. Two milligrams of each sample were placed on the sample holder on the X-ray diffractometer and analyzed. 

Direct Blue 78 (DB78) was used to study the adsorption of TiO_2_-NPs on CS-MTX-NPs. The dye adsorption measurements were conducted by mixing 0.5 g/L of MTX-CS-NPs coated with TiO_2_-NPs with 50 mg/L of DB78 at pH 2. Then, the samples were mixed by magnetic stirrer at 200 rpm for 60 min. The changes in the absorbance at λ_max_ of 600 nm of solution samples were monitored and determined at certain time intervals (0, 10, 15, 20, 25, 30 and 60 min) [43]. The percentage absorption reduction was calculated as follows:

$$\%\;\mathrm{absorption}\;\mathrm{reduction}=\frac{\mathrm{Absorption}\;\mathrm{before}\;\mathrm{adding}\;\mathrm{the}\;\mathrm{NPs}-\mathrm{Absorption}\;\mathrm{after}\;\mathrm{adding}\;\mathrm{the}\;\mathrm{NPs}}{\mathrm{Absorption}\;\mathrm{before}\;\mathrm{adding}\;\mathrm{the}\;\mathrm{NPs}\;}\;\times \;100\%\;$$

### 3.5. In Vitro Drug Release from MTX-CS-NPs Coated with TiO_2_-NPs

The MTX release from uncoated and coated MTX-CS-NPs (F_1_, F_2_ and F_3_) was studied in vitro using dialysis bags. Samples equivalent to 5 mg of MTX were dispersed into 2 mL of phosphate buffer saline (PBS) (pH = 7.4) and put in the dialysis bags and secured. Then, the dialysis bags were immersed in a beaker containing 13 mL of PBS at 37 °C and placed in a shaking water bath (Daihan Scientific, Seoul, Korea) that shook the samples at 100 rpm. Samples of 1 mL from each beaker were withdrawn at certain time points and replaced with 1 mL of fresh PBS kept at 37 °C. Finally, the withdrawn samples were analyzed according to the HPLC-UV method described previously. The drug release was studied in vitro under three different conditions, dark, visible light, and UV illumination. To study the drug release under dark conditions, the room was darkened by turning off the lights and covering the windows completely. On the other hand, under visible light conditions the sun light was allowed to get into the room. For studying the samples under UV illumination, the room was darken and UV lamps (lamp power = 4 W) were used to illuminate the samples at λ of 370 nm and the lamps were above the samples in a distance of 2 cm [12].

### 3.6. Cell Culture

The antitumor activity of MTX, CS, TPP, MTX-CS-NPs and MTX-CS-NPs coated with TiO_2_-NPs (F_1_, F_2_ and F_3_) was studied in vitro using the human breast cancer tumor cell line MCF-7. The MCF-7 cell line was grown in RPMI1640 containing 10% (*v*/*v*) FBS, 100 U/mL penicillin G, and 100 µg/mL streptomycin and incubated at 37 °C, 5% CO_2_ for 24 h [5,44]. MTT viability assay was employed to assess the time-dependent cytotoxicity of the samples. After reaching a proper population with a detachment of 0.25% trypsin in phosphate buffer saline (PBS), cells were seeded in 96-well plates with a cell density of 10^4^ cells/well and allowed to grow for 24 h at the same conditions. For MTX group, the cells were treated with 400 µg/mL of MTX. For F_1_, F_2_ & F_3_, weights of each formulation that contain MTX equivalent to 400 µg/mL were used. For the cells treated with CS or TiO_2_ NPs, the amounts of CS and TiO_2_ NPs were equivalent to those used in preparing F_1_. or using weights that are equivalent to 400 µg/mLof MTX. Equivalent volume of fresh media was added to control cells. After incubation for 24, 48, 72 h, the MTT assay was carried out in triplicate. The old media was replaced by 150 µL of 0.5 mg/mL MTT in PBS in each well. The three plates were covered with aluminum foil and incubated for 4 h. Then the old media was replaced with 150 µL DMSO in order to dissolve the formazan crystals. ELISA plate reader (Synergy™ 2 Multi-Mode Microplate Reader, Biotek, Winooski, VT, USA) was applied to measure the absorbance of each well at 570 nm. 

### 3.7. Statistical Analysis

Statistical analysis was performed using GraphPad^®^ Prism statistical software (version 9.0.0, GraphPad software, San Diego, CA, USA). MTT assay experiments were conducted in triplicates. Results were tested for normality using Shapiro-Wilk test and were normally distributed. Following that, two-way ANOVA was applied to perform concomitant comparison between the variable treatment groups and the effect of treatment duration. A *p*-value of <0.05 was considered as a significant result. All data are represented as mean value ± SD.

## 4. Conclusions

Novel UV-sensitive biodegradable polymeric nanoparticles made of chitosan loaded with methotrexate and functionalized with TiO_2_-NPs were constructed. This system is monodispersed, positively charged with sizes as low as 240 nm and entrapment efficacies reaching 80%. SEM, FTIR, XRD and the dye tests proved the chemical deposition of TiO_2_-NPs on MTX-CS-NPs. The release of the drug from these coated NPs can be triggered by UV light and controlled by the amount of TiO_2_-NPs used in the coatings. This system showed a significant effect against MCF-7 breast cancer cells, with viabilities as low as 7%, in comparison to the pure chitosan, MTX and TiO_2_-NPs. Finally, coating MTX-CS-NPs with TiO_2_-NPs can be used to remotely trigger MTX release depending on patient needs. This is expected to allow a time-controlled release of MTX that may maximize tumor killing and minimize its spread. To assure that, further studies on the safety of the system and its behavior in vivo are to be performed.

## Figures and Tables

**Table 1:** Physical characterization of MTX-CS-NPs before coating and MTX-CS-NPs coated with TiO_2_-NPs (1:1, 1:2 and 2:1).

Formula	Ratio of MTX-CS-NPs to TiO_2_	Size (nm)	PDI	Zeta (mV)	% EE
CS-MTX NPs	Without TiO_2_ NPs	169.00 ± 3.15	0.27 ± 0.04	+9.37 ± 0.35	68.31 ± 0.98
F_1_	1:1	411.93 ± 17.04	0.49 ± 0.01	+24.20 ± 1.41	60.23 ± 1.23
F_2_	1:2	241.13 ± 3.64	0.25 ± 0.04	+26.10 ± 0.70	78.96 ± 1.40
F_3_	2:1	262.27 ± 2.81	0.54 ± 0.07	+25.77 ± 0.76	63.90 ± 2.05

## Data Availability

Data is contained within the article.
